# Discovery of Drug-Like Ligands for the Mac1 Domain of SARS-CoV-2 Nsp3

**DOI:** 10.1177/2472555220960428

**Published:** 2020-09-28

**Authors:** Rajdeep S. Virdi, Robert V. Bavisotto, Nicholas C. Hopper, Nemanja Vuksanovic, Trevor R. Melkonian, Nicholas R. Silvaggi, David N. Frick

**Affiliations:** 1 Department of Chemistry and Biochemistry, The University of Wisconsin–Milwaukee, Milwaukee, WI, USA

**Keywords:** COVID-19, antiviral drug target, macrodomain, coronavirus, thermal shift

## Abstract

Small molecules that bind the SARS-CoV-2 nonstructural protein 3 Mac1 domain in place of ADP-ribose could be useful as molecular probes or scaffolds for COVID-19 antiviral drug discovery because Mac1 has been linked to the ability of coronaviruses to evade cellular detection. A high-throughput assay based on differential scanning fluorimetry (DSF) was therefore optimized and used to identify possible Mac1 ligands in small libraries of drugs and drug-like compounds. Numerous promising compounds included nucleotides, steroids, β-lactams, and benzimidazoles. The main drawback to this approach was that a high percentage of compounds in some libraries were found to influence the observed Mac1 melting temperature. To prioritize DSF screening hits, the shapes of the observed melting curves and initial assay fluorescence were examined, and the results were compared with virtual screens performed using AutoDock Vina. The molecular basis for alternate ligand binding was also examined by determining a structure of one of the hits, cyclic adenosine monophosphate, with atomic resolution.

## Introduction

Direct-acting antivirals (DAAs) are desperately needed to treat COVID-19 patients and stem the devastation caused by the current SARS-CoV-2 pandemic. DAAs are typically developed from potent inhibitors of viral enzymes or high-affinity ligands of viral proteins. For example, remdesivir inhibits the SARS-CoV-2 RNA-dependent RNA polymerase, halting SARS-CoV-2 replication.^1^ Based on past experiences, any effective DAA therapy will likely require a cocktail of more than one antiviral agent because drug resistance evolves rapidly. Methods are therefore needed to rapidly identify small-molecule drug-like ligands for as many SARS-CoV-2 proteins as possible. The ~29,900-nucleotide SARS-CoV-2 genome encodes many potential DAA targets, including 16 nonstructural proteins (nsps), 4 structural proteins, 6 accessory proteins, and possibly many others. Most SARS-CoV-2 nsps are products of the rep1b open reading frame that encodes a short (aka ORF1a) and long (aka ORF1b) polyprotein because an internal RNA hairpin occasionally causes translational frameshifting. The subject of this study is the multifunctional 945-amino acid-long nsp3 protein that cleaves the three junctures separating nsp1, nsp2, and nsp3.^2^ SARS-CoV-2 nsp3 is most likely tethered to the ER with two ubiquitin-like domains (Ubl1 and Ubl2), two papain-like protease domains (PLP1^pro^ and PLP2^pro^), three macrodomains (Mac1, Mac2, and Mac3), a nucleic acid-binding domain, and a hypervariable region facing the cytoplasm.

In a previous study, Frick et al.^3^ characterized the SARS-CoV-2 Mac1 domain (aka the X domain) and its ability to bind ADP–ribose (ADPr). Here, we report the results of pilot screens designed to find drug-like Mac1 domain ligands, which might facilitate DAA design, or which could be useful as molecular probes. A differential scanning fluorimetry (DSF; aka the thermal shift, or ThermoFluor)^4–6^ assay was optimized and used to screen 726 compounds in the National Institutes of Health clinical collection (NIHcc), the National Cancer Institute (NCI) mechanistic set (540 compounds), and Sigma-Aldrich’s 1280-compound Library of Pharmacologically Active Compounds (LOPAC^1280^). Since up to 5% of compounds in each set influenced the apparent melting temperature, compounds were prioritized using information derived from individual melting curves and virtual screens performed with AutoDock Vina.^7^ A high-resolution structure of one hit compound, cyclic adenosine monophosphate (cAMP), was determined in complex with SARS-CoV-2 Mac1, revealing at atomic resolution the capacity of the binding cleft to accommodate other ligands.

## Materials and Methods

Purified SARS-CoV-2 Mac1 protein was prepared as described previously.^3^ DSF assays were performed in 96-well PCR plates using an Eppendorf Mastercycler ep Realplex Quantitative Realtime PCR System, with each well containing 19 µL of master mix (5 µM Mac1) and 1 µL of a compound stock (10 mM for screening) or DMSO. The master mix was prepared by adding 20 µL of 500 µM Mac1 and 2.5 µL of 5000× SPYRO Orange protein gel stain (Sigma-Aldrich cat. S5692) to 1977.5 µL of buffer (20 mM MOPS, 25 mM NaCl, pH 7). The 96-well PCR plate was then sealed by a clear adhesive film and centrifuged at 1100 rpm for 5 min. The temperature was raised from 20 to 95 °C at a rate of 2 °C/min while measuring the fluorescence in the “TAMRA” channel. Each plate included both negative (DMSO) and positive (ADPr) controls. *T_m_* values were calculated by fitting the data to eq 1 using either GraphPad Prism (GraphPad, La Jolla, CA) or TSA-CRAFT (https://sourceforge.net/projects/tsa-craft/).^8^

(1) $$F_{obs}\left(T\right)=F_{min}\frac{F_{max}-F_{min}}{1+e^{\left(\frac{T_{m}-T}{a}\right)}}$$

In eq 1, *F_obs_* (*T*) is the observed fluorescence at each temperature (*T*), *F_min_* is the minimum observed fluorescence, *F_max_* is maximum observed fluorescence, and *a* is a Hill slope. Two methods were used to estimate the affinity of Mac1 from DSF. First, the observed melting temperatures were plotted versus ligand and fit to eq 2 to determine the amount of compound needed to cause a change in the melting temperature of 50% (EC_50_), and a nonlinear regression was used to estimate ΔT_m max_ (the maximum change in *T_m_*) from the melting temperature of Mac1 in the absence of ligand (*T_m_*_0_):

(2) $$T_{m\;\;obs}=\frac{\Delta T_{m\;\;max}*\left[L\right]}{EC_{50}+\left[L\right]}+T_{m\;\;0}$$

The dissociation constant (*K_d_*) of Mac1 and ADPr and the equilibrium constant describing protein unfolding (*K_u_*) were also estimated using isothermal analysis as described by Bai et al.^9^ Briefly, normalized melting curves were used to calculate the fraction of protein unfolded at a particular temperature (*f_u_*) and those values fitted to the total ligand (*L_t_*) and protein (*P_t_*) concentrations using nonlinear regression and eq 3:

(3) $$f_{u}=\frac{1}{1+\left(\frac{1}{K_{u}}\left(1+\frac{L}{K_{d}}\right)\right)}$$

where

$$L=\frac{\begin{array}{l} \left(L_{t}-P_{t}-K_{d}\left(1+K_{u}\right)\right)+\\ \sqrt{{\left(P_{t}-L_{t}+K_{d}\left(1+K_{u}\right)\right)}^{2}+4L_{t}K_{d}\left(1+K_{u}\right)} \end{array}}{2}$$

Computational ligand screening was performed using both the unligated (6WEY and 6VXS) and ADPr-bound (6W02) forms, using the program AutoDock Vina.^7^ The protein files were downloaded directly from the Protein Data Bank (PDB) and processed as described below before submitting for screening. All solvent molecules (HETATM) were removed from the files. Polar hydrogen atoms were added and Kollman charges were included in the protein files. The converted protein and ligand file pdbqt libraries were uploaded to a parallel computing cluster and run with the following parameters: energy difference = 4; number of recorded modes = 20; and exhaustiveness was set to 12. The docking box location was configured prior to using AutoDock tools. After the docking calculation was complete, the locations, orientations, and binding affinities of the top candidates were examined using UCSF Chimera and tabulated for comparison.

Mac1 was prepared for crystallization as described before^3^ with the following modifications. First, the plasmid was modified to express one additional N-terminal residue (E206) and C-terminus was shortened by three residues such that it encoded residues 206–374 of the SARS-Cov-12 nsp3. After purification and TEV protease cleavage, the tag-free protein was concentrated to 20 mg/mL in 20 mM HEPES, pH 7.5, 150 mM NaCl, and 1 mM TCEP. Crystallization was accomplished as described for the Mac1·AMP complex^10^ (1 µL of concentrated Mac1 was mixed with 1 µL of 30% PEG 4000 and 0.1 M MES, pH 6.5). Plate-shaped crystals grew in 3–7 days at 22 °C.

The Mac1·cAMP complex was prepared by soaking the crystal in a solution containing 35% PEG 4000 and 20 mM cAMP for 30 min. Cryoprotection was accomplished by briefly soaking the crystal in 35% PEG 4000, 20 mM cAMP, and 20% glycerol before plunging it into liquid nitrogen. Diffraction data were collected on Life Sciences Collaborative Access Team (LS-CAT) beamline 21-ID-F at the Advanced Photon Source of the Argonne National Laboratory, which is fitted with a fixed-wavelength beam at 0.97872 Å and a MarMosaic M300 detector. The data were collected with an oscillation width of 0.5° per image for a total oscillation of 180°. The data were processed using HKL2000;^11^ data collection statistics are provided in **Supplemental Table S1**.

The structure was determined by molecular replacement in PHASER^12^ using PDB ID 6WEY,^3^ with solvent molecules and B-factor information removed, as the search model. The model underwent iterative rounds of (re-)building in COOT^13^ and refinement in PHENIX.refine.^14,15^ Translation–libration–screw (TLS) refinement provided a more realistic treatment of the atomic displacement parameters; TLS groups were identified by phenix.find_tls_groups. Model refinement and validation statistics are provided in **Supplemental Table S1**. The coordinates were deposited in the PDB (accession code 7JME).

## Results

### Optimized DSF Assay for SARS-CoV-2 Mac1

**Figure 1.**
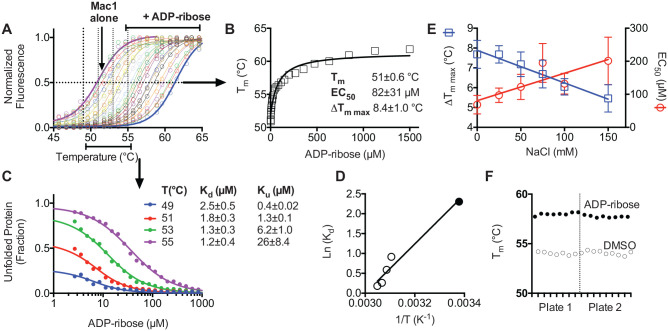
DSF assay optimization. (**A**) Normalized SPYRO Orange fluorescence in the presence of 4.75 µM Mac1 protein at various temperatures in the presence of indicated concentrations of ADPr. Data are fit to eq 1 using nonlinear regression with GraphPad Prism. (**B**) *T_m_* values obtained from direct fitting to eq 1. Data are fit to eq 2. (**C**) Isothermal analysis^9^ of percent unfolded protein at each indicated temperature. Data are fit to eq 3 with indicated constants. Uncertainties are standard errors of the curve fits. (**D**) Van’t Hoff plot of estimated *K_d_* values from **C** (open circles) and the *K_d_* for ADPr binding to Mac1 that was previously determined at 23 °C using isothermal titration calorimetry (filled circle).^3^ (**E**) ADPr titrations in 20 mM MOPS pH buffer supplemented with indicated NaCl concentrations. Plotted are best-fit Δ*T_m max_* (squares, left *y* axis) and EC_50_ (circles, right axis). Error bars mark standard errors in the curve fits. (**F**) *T_m_* values for positive (500 µM ADPr) and negative (DMSO only) controls from two different 96-well plates (Z′ factor = 0.72).

DSF has been used previously to study ligand binding to viral macrodomains.^16,17^ In DSF experiments using SARS-CoV-2 Mac1, the presence of ADPr raised the Mac1 melting temperature in a concentration-dependent manner (**Fig. 1A**). To estimate the ligand concentrations needed to alter melting temperatures by 50% (EC_50_), melting temperatures were fit to eq 2 (**Fig. 1B**). Such EC_50_ values do not, however, describe protein–ligand affinity because DSF assays do not directly measure binding. The isothermal analysis recently described by Bai et al.^9^ was therefore used to estimate binding affinities. Fits of the fraction of protein unfolded at various temperatures in the presence of various ligand concentrations (**Fig. 1C**) were used to estimate a dissociation constant (*K_d_*) and equilibrium unfolding constant (*K_u_*). Each depended on temperature (**Fig. 1D**), and when fit to the Van’t Hoff relationship, the values were in good agreement with the dissociation constant describing the interaction of ADPr and Mac1 (10 µM) determined at 23 °C.^3^

**Figure 2.**
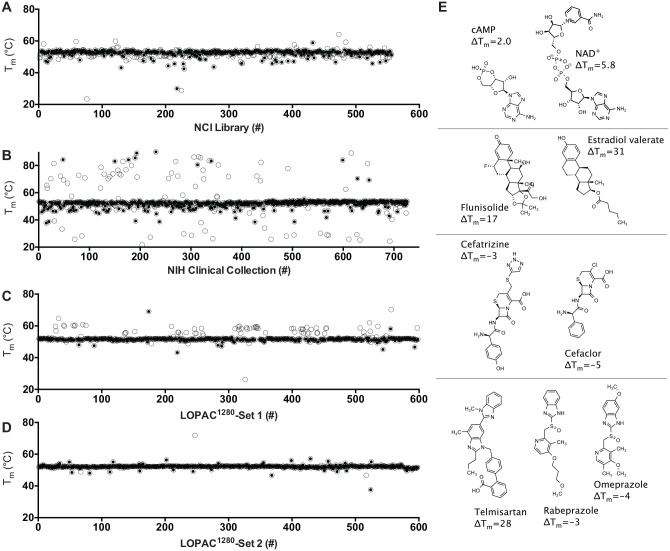
DSF screens of FDA-approved drugs and drug-like compounds for SARS-CoV-2 Mac1 ligands. *T_m_* values calculated by fitting melting curves to eq 1 (open circles) obtained for Mac1 in the presence of each compound in (**A**) the NCI library, (**B**) the NIHcc, and (**C,D**) the LOPAC^1280^. Assays yielding a “typical” melting curve, as defined by the TSA-CRAFT algorithm, are noted (filled circles). (**E**) Selected hit compounds separated based on chemotype: nucleotides, steroids, β-lactam antibiotics, and benzimidazoles.

DMSO did not change the melting curve even at concentrations as high as 10% (v/v), and similar results were also obtained when titrations with ADPr were repeated in various buffers, with the pH ranging from 6.5 to 8.0, or in the presence of various concentrations of divalent metal cations (Mg^2+^ or Mn^2+^). In contrast, the ionic strengths of the assay buffers influenced the results, with the largest Δ*T_m_* values and lowest EC_50_ values being obtained at the lowest ionic strengths (**Fig. 1E**). Based on these results, DSF assays were subsequently performed in 20 mM MOPS buffer, pH 7, containing 25 mM NaCl to reduce possible nonspecific interactions with ligands. Z′ factors^18^ were always above 0.5 for each plate and typically above 0.7. Plate-to-plate variability was negligible (**Fig. 1F**). The first screen was performed using compounds from an NCI library (https://dtp.cancer.gov/repositories/) (**Fig. 2A**),^19^ the second was performed using the NIHcc (https://commonfund.nih.gov/molecularlibraries/tools) (**Fig. 2B**),^20^ and the third using Sigma-Aldrich’s LOPAC^1280^ (**Fig. 2C,D**). Although ligands that reduce a protein’s *T_m_* are often assumed to bind and stabilize unfolded structures,^21^ we nevertheless also examined some of these hits in more detail. In addition to nucleotides suspected to bind in place of ADPr, other noteworthy hits relevant to current COVID-19 research included the angiotensin-converting enzyme (ACE) inhibitor telmisartan, several steroids, and β-lactam antibiotics (**Fig. 2E**).

### Prioritizing Hits in DSF Screens

**Figure 3.**
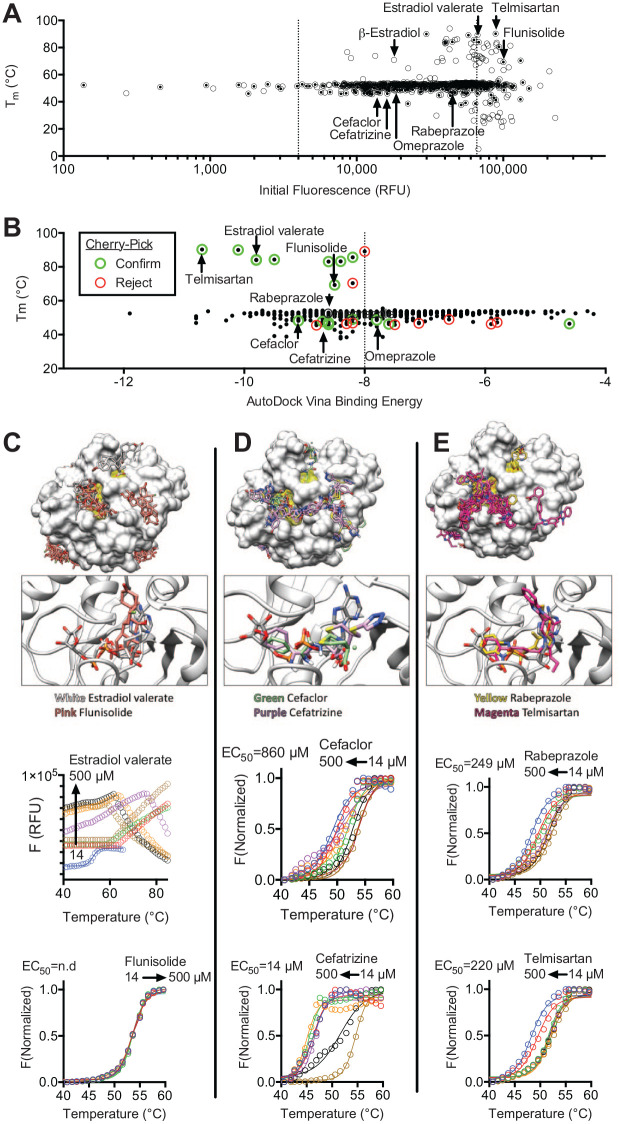
Methods to prioritize hits from DSF screens. (**A**) Plot of *T_m_* values for samples in the NIHcc plotted versus fluorescence observed at the beginning of each melt (i.e., at 20 °C). “Typical” melting curves are highlighted (filled circles). The dotted lines are arbitrary cutoffs drawn at three times more and less than the average fluorescence intensity recorded in all assays. (**B**) AutoDock Vina binding energies obtained for each compound after docking with PDB file 6WEY (*y* axis) compared with the *T_m_* derived using TSA-CRAFT. (**C–E**) Representative structures obtained using AutoDock Vina Virtual Screening. The top panels show the top 20 binding modes for selected compounds, and the bottom panels show the top binding mode for each compound compared with the ADPr bound in PDB file 6W02. The ADPr-binding cleft is highlighted in yellow. The concentration–response analysis of each compound in DSF assays is shown, along with EC_50_ values.

Screening results reveal that the major limitation of this approach is that a high percentage of compounds in some libraries influence the observed protein melting temperature, which was particularly evident with the NIHcc. Many of these compounds either quench fluorescence, fluoresce themselves, or interact with the reporter dye. To exclude such compounds in follow-up experiments, the TSA-CRAFT software package was used to identify what it defines as “typical” curves (**Figs. 2 and 3**, filled circles). We found that such interfering compounds could also be identified by simply plotting *T_m_* values versus the initial fluorescence seen in the melting curve (**Fig. 3A**).

As another method for hit prioritization, “virtual” screens were performed with SARS-CoV-2 Mac1 crystal structures (PDB files 6WEY, 6W02, and 6VXS) as targets in the program AutoDock Vina (**Fig. 3A**). Each was searched free from ligands. Binding sites were not restricted but, for most compounds, minimum binding energy (best-fit) values were obtained for structures in which the compound docked near the ADPr-binding site. Plots of AutoDock Vina scores versus *T_m_* could be used to identify compounds for follow-up assays (**Fig. 3B**). First, there was a clear correlation between the number of hits and AutoDock Vina scores, with more hits clustering at lower energies. Second, when cherry-picking assays were performed on hits, those with lower energy scores (12/15) were more likely to be reproducible than compounds with higher energy scores (3/9) (**Fig. 3B**). Close examination of molecular models generated by AutoDock Vina revealed that the steroids (**Fig. 3C**), β-lactams (**Fig. 3D**), and benzimidazoles (**Fig. 3E**) could each occupy the ADPr-binding cleft on SARS-CoV-2 Mac1. The larger compounds in each class make more contacts with amino acids in the cleft, explaining their higher binding energies.

By combining these prioritization methods, compounds that more likely bind Mac1 could be differentiated from those that likely do not. For example, all nucleotides that were hits yielded results similar to those seen with ADPr. However, the steroids either yielded atypical curves (β-estradiol) or increased the DSF assay fluorescence (estradiol valerate and flunisolide). When new aliquots of selected compounds were purchased, abnormal melting curves were observed with estradiol valerate and no effects were observed with flunisolide (**Fig. 3C**). In contrast, fresh batches of both lactams and two benzimidazoles yielded the same effects seen with the screening library (**Fig. 3D,E**). Interestingly, fresh telmisartan yielded a different effect, lowering the apparent *T_m_*, as was seen with related compounds (**Fig. 3D**), suggesting that a possible degradation product led to the *T_m_* increase observed using the library sample.

### Structure of cAMP Bound to SARS-CoV-2 Mac1

**Figure 4.**
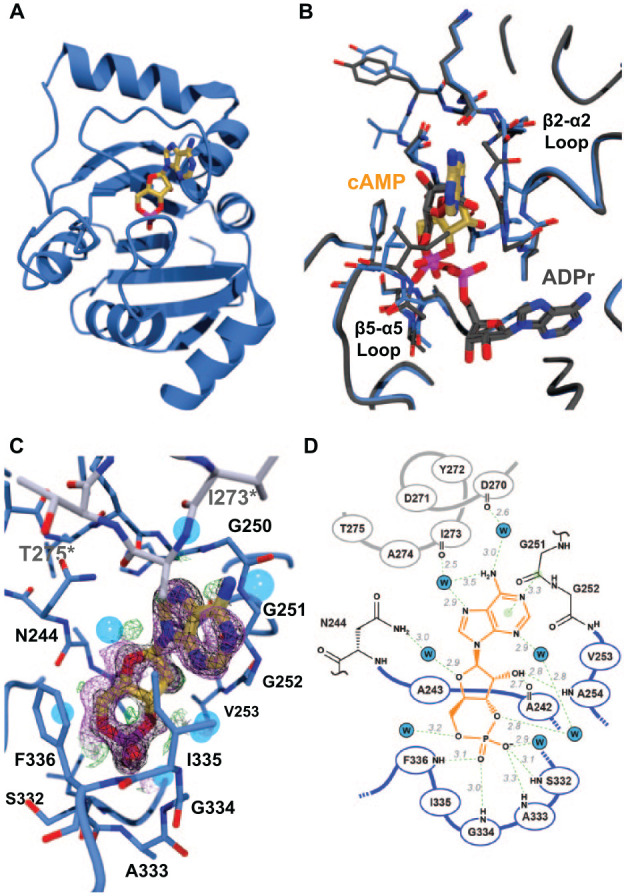
The Mac1 protein binds cAMP in an unexpected way. (**A**) Ribbon diagram showing the SARS-CoV-2 MaC1 domain with bound cAMP (gold sticks). (**B**) Overlay of the structures of the Mac1 domain with bound cAMP (gold sticks) or ADPr (gray sticks). The β2-α2 and β5-α5 loops are noted for reference. Note the difference in conformation of the β2-α2 loop (the section carrying G251). The reorientation of this loop allows it to pack against the adenine base of cAMP. (**C**) cAMP-binding site. The Mac1 domain protein is shown as a blue Cα trace with important residues shown as thin blue sticks. Water molecules are shown as transparent blue spheres. The stretch of amino acids shown in gray sticks represents the symmetry-related Mac1 molecule that makes contact with cAMP. The simulated annealing composite omit map is shown as a magenta mesh contoured at 1.0σ, the 2Fo-Fc map is shown in black, also at 1.0σ, and the Fo-Fc map is shown as green and red mesh at +3.0σ and –3.0σ, respectively. (**D**) Schematic representation of the Mac1·cAMP complex showing polar interactions between the enzyme and ligand. The cAMP ligand is shown with orange bonds. The heavy blue lines and residues drawn with black bonds represent the Mac1 protein. The heavy gray line represents the symmetry-related molecule that makes solvent-mediated contacts with cAMP. Solvent molecules are shown as blue circles with a W. Potential hydrogen bonding interactions are shown as dashed green lines with the associated distances in gray italics. The stacking interaction described in the text with the G251–G252 peptide bond is shown as a green line with light green circles at each end.

The protein construct used to determine the structure of the apoenzyme (PDB ID 6WEY; nsp3 residues 207–377) crystallized with such tight packing that it proved impossible to obtain structures of the ligand-bound Mac1 protein. Thus, we were in the unusual position of trying to get looser packing and poorer resolution. Adding a single N-terminal residue to the protein (nsp3 residues 206–374) was enough to change the packing from orthorhombic (P2_1_2_1_2_1_) to monoclinic (P2_1_). To verify binding, each of the compounds in **Figure 2** was both co-crystallized with this new Mac1 construct and soaked into crystals of the apoprotein. Despite considerable effort, we were only able to obtain a complex structure with cAMP. This model contains one molecule of Mac1 in the asymmetric unit, comprising 166 amino acids (residues 208–373), 135 water molecules, and 1 molecule of cAMP. The cAMP binds in the cleft between the β2-α2 loop (residues K248–V253) and the β5-α5 loop (residues L331–D339); the bottom of this cleft is formed by strand β2 (**Fig. 4A–C**). The electron density is well defined for all but the solvent-exposed edge of the adenine base, which appears to be wobbling at the brink of the binding site. The interactions with Mac1 are comprised entirely of hydrogen bonding interactions to the main chain, particularly in the β5-α5 loop (**Fig. 4D**). The adenine base is held only by water-mediated interactions to the β2-α2 and β5-α5 loops (e.g., the amide nitrogen atoms of V253 and I335) and a stacking interaction with the G251–G252 peptide bond. There are also two water-mediated contacts with a symmetry-related Mac1 molecule (**Fig. 4D**). Since cAMP makes no close contacts to this symmetry mate, we do not believe that the proximity of the neighboring Mac1 molecule significantly influences the binding pose of the ligand. The 2′ hydroxyl group of cAMP makes hydrogen bonding interactions with the carbonyl group of A242 on strand β2 and the amide group of A254 on helix α2 (**Fig. 4D**). On the other side of the ribose ring, O4′ interacts with the side chain of N244 through the intercession of the water molecule. The 3′ and 5′ oxygen atoms of the ribose moiety interact, through water, with the amide of I335 and the carbonyl of A243, respectively. Given the density of interactions with the Mac1 protein, the two phosphate oxygen atoms are likely the main drivers of cAMP binding. The β5-α5 loop forms a string of amide groups that lock the phosphate of cAMP in place with hydrogen bonding interactions to these two oxygen atoms.

The binding mode of cAMP was compared with those of ADPr (PDB ID 6W02;^10^ **Fig. 4C**) and AMP (PDB ID 6W6Y;^10^ not shown). Chain A of 6W02 was superimposed onto the Mac1·cAMP model using the SSM algorithm^22^ as implemented in COOT. The two models were fit with a root mean square deviation (RMSD) of 0.41 Å for all 166 Cα atoms in the Mac1·cAMP model. Overlaying the AMP complex structure gave an RMSD of 0.40 Å for all Cα atoms. These low RMSD values indicate that the structures are identical in gross terms, with only small differences in the orientations of small portions, such as surface loops, as one would expect when comparing multiple structures of the same protein. What is interesting is that whereas the common portions of ADPr and AMP overlay almost perfectly, cAMP binds such that the cyclic phosphate matches with the β-phosphate of ADPr, the ribose moiety corresponds to the terminal ribose of ADPr, and the adenine base is directed toward solvent. Consequently, there is no overlap at all of cAMP and AMP. This was entirely unexpected, since the adenine bases in the ADPr- and AMP-bound Mac1 domain structures are very solidly bound, with strong electron density and low B factors. The only possible explanation for this is that the geometry of the cyclic phosphate moiety, particularly its relationship to the ribose ring, does not comport well with the α-phosphate-binding site of Mac1 and is instead a better fit for the β-phosphate/terminal ribose-binding site. This alternative binding pose results in slight reorientations of the β2-α2 and β5-α5 loops (**Fig. 4C**), which move away from each other to accommodate the adenine base of cAMP. It is also intriguing that, if the cAMP in this model were joined to the AMP in 6W6Y by a phosphodiester bond (and the P-O3′ bond in cAMP were broken), the result would be reminiscent of diadenosine 5′,5′-diphosphate, or related compounds like NAD(H).

## Discussion

The idea that SARS-CoV Mac1 functions as an enzyme in the cell to remove ADPr from antiviral proteins suggests that Mac1 might be an important new drug target for COVID-19.^23^ A thermal shift binding assay was therefore developed to facilitate those efforts. The main drawback with the DSF assay was the high percentage of hits for some libraries. Various methods to successfully prioritize hits are described above, with the simplest being an examination of the initial fluorescence values in DSF assays (**Fig. 3A**). DSF’s other main disadvantage is that it requires relatively large amounts of protein, but this is a minor concern because of the ease with which Mac1 can be produced from *Escherichia coli*.^3^ The most attractive alternative to a DSF binding assay would be an enzyme assay that monitors the ability of Mac1 to hydrolyze ADPr-based substrates, which are presently under development.^24^

The most intriguing hits in DSF screens were the steroids and telmisartan (**Fig. 3**). Unfortunately, closer examination revealed that the steroid effects in DSF appeared to be artifacts. Caution should also be exercised because telmisartan and similar compounds lowered the apparent *T_m_* of SARS-CoV-2 Mac1. This could mean they bind to the protein’s unfolded state, but it is worth noting that similar destabilizing compounds were found to inhibit macrodomains in assays not based on thermal shifts,^24^ suggesting that the destabilizing compounds might bind a folded protein that assumes a different conformation.

The idea that SARS-CoV-2 ADPr-binding cleft can accommodate other ligands is supported by the x-ray structure of Mac1-bound cAMP. Surprisingly, the adenine base of cAMP does not bind in the adenine-binding cleft identified in the structures of Mac1 bound to ADPr or AMP.^10^ Instead, cAMP binds in the site occupied by the β-phosphate/terminal ribose unit of ADPr. This result underscores the importance of computational modeling and experimental structure determination in assessing the hits from high-throughput screening campaigns. Based on this crystal structure, it is likely that scaffolds containing a central phosphate (or diphosphate) or sulfate group could be expected to bind to this same ADPr-binding cleft. The interaction between Mac1 and cAMP, which binds Mac1 with a similar affinity as ADPr,^3^ points to other possible biological roles for Mac1 and hints that cyclic mono- or dinucleotide second messengers might allosterically modulate other nsp3 activities.

The next step in this project will be to examine the effect of promising antiviral compounds on cells harboring SAR-CoV-2 or surrogate reporter viruses. Some of the compounds above might inhibit SARS-CoV-2 replication based on the fact that Shimizu et al. showed that small molecules found in virtual screens targeting the homologous nsp3 macrodomain from Chikungunya virus inhibit replication of Chikungunya replicons.^25^ However, further chemical optimization would most likely be necessary for these probes to be useful in cellular studies. Fortunately, many hits reported here are already Food and Drug Administration (FDA)-approved drugs with hundreds of analogs available to facilitate such work. Alternatively, this optimized DSF assay could be used to screen larger, more diverse libraries for more attractive probe candidates.

## Supplemental Material

Supplemental_Information – Supplemental material for Discovery of Drug-Like Ligands for the Mac1 Domain of SARS-CoV-2 Nsp3 Click here for additional data file. Supplemental material, Supplemental_Information for Discovery of Drug-Like Ligands for the Mac1 Domain of SARS-CoV-2 Nsp3 by Rajdeep S. Virdi, Robert V. Bavisotto, Nicholas C. Hopper, Nemanja Vuksanovic, Trevor R. Melkonian, Nicholas R. Silvaggi and David N. Frick in SLAS Discovery
