# Supplementary material for: Discovery of Drug-Like Ligands for the Mac1 Domain of SARS-CoV-2 Nsp3
Source: SLAS Discov. 2020 Sep 28;25(10):1162–70. doi: 10.1177/2472555220960428 (PMC7684785; doi:10.1177/2472555220960428)
Supplement: Supplemental_Information – Supplemental material for Discovery of Drug-Like Ligands for the Mac1 Domain of SARS-CoV-2 Nsp3 [file Supplemental_Information.pdf]

Supplemental Information

Discovery of Drug-like Ligands for the Mac1 Domain of SARS-CoV-2 Nsp3

Rajdeep S. Virdi, Robert V. Bavisotto, Nicholas C. Hopper, Nemanja Vuksanovic, Trevor R. Melkonian, Nicholas R. Silvaggi & David N. Frick*

Table S1. Crystallographic data collection and model refinement statistics for the SARS-CoV-2 Mac1 domain in complex with cAMP.

**Data collection**	
Resolution range (Å) (last shell)[a]	24.70 - 1.55 (1.61 - 1.55)
Space Group	P 2_1
a, *b*, *c* (Å)	37.2, 33.0, 60.5
α, β, γ (°)	90.0, 96.4, 90.0
R_{merge}[a]	0.076 (0.291)
R_{meas}[a]	0.091 (0.344)
R_{pim}[a]	0.049 (0.182)
$CC_{1/2}$[a]	0.992 (0.891)
No. of unique reflections[a]	20508 (1938)
Completeness (%)[a]	95.05 (90.9)
Multiplicity[a]	3.4 (3.3)
$\langle I/\sigma(I)\rangle$[a]	10.9 (4.2)
**Model Refinement**	
Reflections used in refinement	20433 (1938)[b]
Reflections used for R_{free}	1998 (189)
R_{cryst} (R_{free})	0.157 (0.182)
Average B factor (Å^2)	20.3
Protein atoms	19.1
Solvent	32.0
Ligand	17.2
Root-mean-square (RMS) deviations	
Bond lengths (Å)	0.008
Bond angles (°)	0.893
Coordinate error (Å)	0.11
Ramachandran statistics	
Favored/allowed/outliers (%)	99.4/0.6/0.0
Rotamer outliers (%)	0.8
Clashscore	0.8

[a]Values in parentheses apply to the high-resolution shell indicated in the resolution row

[b]The limits of the high-resolution bin for refinement were 1.59 – 1.55Å
